# Simultaneous Determination of 34 Amino Acids in Tumor Tissues from Colorectal Cancer Patients Based on the Targeted UHPLC-MS/MS Method

**DOI:** 10.1155/2020/4641709

**Published:** 2020-08-01

**Authors:** Yang Yang, Feng Zhang, Shouhong Gao, Zhipeng Wang, Mingming Li, Hua Wei, Renqian Zhong, Wansheng Chen

**Affiliations:** ^1^Department of Pharmacy, Changzheng Hospital, The Second Military Medical University of CPLA, Shanghai 200003, China; ^2^Department of Pharmacy, The 71st Group Army Hospital of CPLA Army, The Affiliated Huaihai Hospital of Xuzhou Medical University, Xuzhou 221004, China; ^3^Department of Laboratory Diagnostics, Changzheng Hospital, The Second Military Medical University of CPLA, Shanghai 200003, China

## Abstract

A targeted ultrahigh-performance liquid chromatography-tandem mass spectrometry (UHPLC-MS/MS) method was established and validated for the simultaneous determination of 34 amino acids in tissue samples from colorectal cancer (CRC) patients. The chromatographic separation was achieved on an Agilent ZORBAX SB-C_18_ column (3.0 × 150 mm, 5 *μ*m) with a binary gradient elution system (A, 0.02% heptafluorobutyric acid and 0.2% formic acid in water, v/v; B, methanol). The run time was 10 min. The multiple reaction monitoring mode was chosen with an electrospray ionization source operating in the positive ionization mode for data acquisition. The linear correlation coefficients were >0.99 for all the analytes in their corresponding calibration ranges. The sample was pretreated based on tissue homogenate and protein precipitation with a 100 mg aliquot sample. The average recovery and matrix effect for 34 amino acids and 3 internal standards were 39.00%∼146.95% and 49.45%∼173.63%, respectively. The intra- and interday accuracy for all the analytes ranged from −13.52% to 14.21% (RSD ≤8.57%) and from −14.52% to 12.59% (RSD ≤10.31%), respectively. Deviations of stability under different conditions were within ±15% for all the analytes. This method was applied to simultaneous quantification of 34 amino acids in tissue samples from 94 CRC patients.

## 1. Introduction

**Figure 1:**
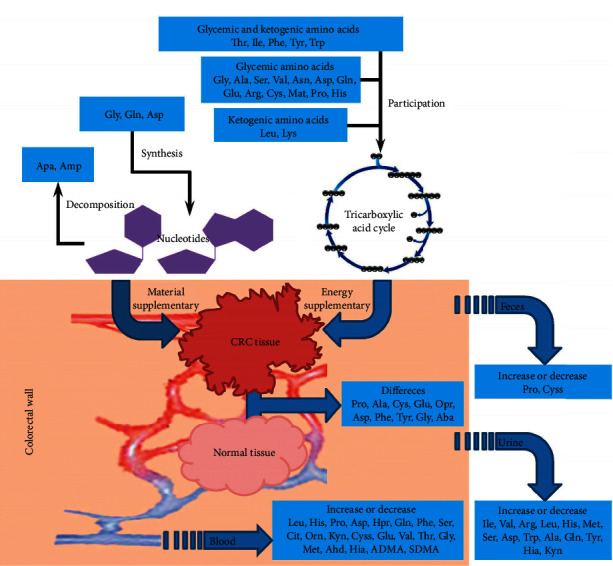
The association of amino acids with the formation, growth, and metabolism of CRC.

Amino acids play an essential role in both metabolism and proteome. Proper amino acid management is critical for maintaining cell function and organismal viability, particularly under metabolic disturbance. With the alterations in tumor cell metabolism recognized as a hallmark of cancer, there have been more research studies on amino acids, which are involved in the primary tumor formation, growth, and metabolism. As shown in Table 1 [1–18], studies in the field of colorectal cancer (CRC) metabolism were routinely performed to elucidate the difference of amino acid profiles in blood, urine, and feces between CRC patients and healthy volunteers or between CRC tissue and normal tissue. Recent findings are related to (1) glycemic and/or ketogenic amino acids that are involved in the energy metabolism by entering tricarboxylic acid cycle pathway; (2) amino acids that are synthetic precursors of pyrimidine bases or provide nitrogen sources for the synthesis of purine bases; and (3) amino acids that are the decomposition products from cytosine, uracil, and thymine nucleotides and that can reflect the severity of malignant tumor and the therapeutic effect of chemotherapy for malignant tumor [19]. To sum up, details about the association of amino acids with the formation, growth, and metabolism of CRC are presented in Figure 1. Besides, as a higher level of sarcosine was observed in the urine of patients with prostate cancer [20–22], sarcosine was believed to be closely related to cancer and also investigated in our study.

Despite the overlap between the results of the targeted and nontargeted metabolomic assays, there were also substantial inconsistencies. Whenever the assessment of a specific pathway such as amino acids became the focus of interest, a targeted metabolomic assay would seem preferable to a nontargeted one [23]. Generally, gas chromatography-mass spectrometry (GC-MS) was available for the amino acid analysis in some laboratories [24–26], but it required a derivatization step for nonvolatile analytes, which was important for the analyte detection and quantification, but could be avoided when a liquid chromatography-mass spectrometry (LC-MS) method was adopted. Amino acids, as a group of polar chemical compounds, should also be modified by using a derivatization reagent to make them volatile and thus accessible for GC-MS [27]. Over the past two decades, many researchers had described the ultrahigh-performance liquid chromatography-tandem mass spectrometry- (UHPLC-MS/MS-) based method for a targeted metabolomic study with increasing levels of sophistication [28–30]. Out of these considerations, thirty-four endogenous amino acids in human biological samples were involved in our study by using the LC-MS technique.

Amino acid metabolomics in human serum has been studied on a larger scale due to its potential diagnostic value in patients with breast, lung, ovarian, head and neck, gastric, and pancreatic cancers or in CRC patients [31], suggesting that the amino acid profiling in plasma/serum or in other body fluids or selected tissue samples might be a new tool for early diagnosis of cancers [32]. As different cancer subtypes show distinct metabolic phenotypes, the present paper aimed to evaluate the amino acid profile in clinical samples from CRC patients by means of a targeted metabolomic assay based on a UHPLC-MS/MS method, which was established and validated for the quantitative analysis of 34 amino acids in tumor tissues. So far, no research has ever evaluated the ability of the amino acid metabolic phenotype of CRC patients. Additionally, this was the first experiment to show the amino acid profile differences between cancerous, paracancerous, and normal tissue of CRC patients, which may shed light on the metabolic stability of amino acids in humans and provide biological information to find and evaluate a new diagnostic tool for the further study of CRC.

## 2. Materials and Methods

### 2.1. Chemicals and Reagents

Thirty-four amino acids, including glycine (Gly), L-alanine (Ala), L-serine (Ser), L-valine (Val), L-threonine (Thr), L-leucine (Leu), L-isoleucine (Ile), L-aspartic acid (Asp), L-lysine (Lys), L-glutamine (Gln), L-glutamic acid (Glu), L-phenylalanine (Phe), L-arginine (Arg), L-tyrosine (Tyr), L-proline (Pro), L-asparagine (Asn), L-methionine (Met), L-tryptophan (Trp), L-cysteine (Cys), L-histidine (His), L-citrulline (Cit), asymmetric dimethylarginine (ADMA), L-cystine (Cyss), sarcosine (Sar), *β*-alanine (3-aminopropanoic acid, Apa), *β*-aminoisobutyric acid (3-amino-2-methylpropanoic acid, Amp), *γ*-aminobutyric acid (4-aminobutyric acid, Aba), 5-oxo-L-proline (Opr), 4-hydroxy-L-proline (Hpr), L-ornithine (Orn), 2-amino-L-hexanoic diacid (*α*-aminoadipic acid, Ahd), hippuric acid (Hia), symmetric dimethylarginine (SDMA), and L-kynurenine (Kyn) were purchased from the National Institutes for Food and Drug Control of China (Beijing, China), Dalian Meilun Biotech Co., Ltd., (Dalian, China), Shanghai Yuanye Biotech Co., Ltd., (Shanghai, China), and Sigma-Aldrich LLC. (Darmstadt, Germany). L-Alanine-d4 (Ala-d4), L-methionine-d3 (Met-d3), and L-phenylalanine-d5 (Phe-d5) were chosen as internal standards (ISs), which were all provided by Toronto Research Chemicals Inc., (Toronto, Canada). The information about the 34 standards and 3 ISs is shown in Supplementary Material Table S1.

Methanol and acetonitrile of HPLC grade were purchased from Merck Inc., (Darmstadt, Germany). Phosphate-buffered solution (PBS) (10x, namely, 0.1 mol/L) of the cell culture grade (Lot. MA0016-May-19B) was purchased from Dalian Meilun Biotech Co., Ltd., (Dalian, China). Heptafluorobutyric acid (HFBA) (Lot. P11933) was obtained from Adamas Reagents Co., Ltd., (Basel, Switzerland). Formic acid (FA) (Lot. C10009619) was gained from Shanghai Macklin Biochemical Co., Ltd., (Shanghai, China). Hydrochloric acid (HCl) of analytical grade was the product of Sinopharm Chemical Reagent Co., Ltd., (Shanghai, China). Sodium chloride injection (0.9% saline, 1000 ml/bag, Lot. 160613) was produced by the pharmaceutical preparation factory of Shanghai Changzheng Hospital, the Second Military Medical University (Shanghai, China). Water was deionized and further purified by a Milli-Q Plus water purification system (Darmstadt, Germany) in our laboratory. The other reagents and solvents were of analytical grade.

### 2.2. Liquid Chromatography and Mass Spectrometry Conditions

An Agilent 1290 UHPLC coupled to an Agilent 6460 triple-quadrupole tandem mass spectrometer (Agilent Inc, USA) was used to establish the method. Chromatographic separation was performed on an Agilent ZORBAX SB-C_18_ column (3.0 × 150 mm, 5 *μ*m), whose temperature was maintained at 50°C. Binary gradient elution was used by mixing the mobile phase A (0.02% HFBA and 0.2% FA in water, v/v) and B (methanol) at a flow rate at 0.4 mL/min. The mobile phase elution procedure was as follows (A/B, v/v): 0 min, 98 : 2; 1 min, 85 : 15; 4 min, 85 : 15; 5 min, 80 : 20; and 10 min, 20 : 80. The run time was 10 min for each sample, and the post time was set at 4.0 min to equilibrate the column pressure. The autosampler temperature was maintained at 4°C. The injected volume was 2 *μ*L with needle wash.

The ionization of analytes was performed based on an electrospray ionization (ESI) source under the positive ionization mode, and the data acquisition was carried out in a multiple reaction monitoring (MRM) mode. Optimized mass spectrometer conditions were as follows: capillary voltage, 5.0 kV; dwell time, 40 ms; collision gas (high-purity nitrogen) pressure, 0.2 MPa; and nebulizer gas (nitrogen) pressure, 50 psi. The dry gas temperature was 325°C and delivered at 10 L/min. The sheath gas temperature was 350°C at the flow rate of 12 L/min.

All data were acquired and processed using Agilent Mass Hunter workstation software (version B.07.00). The optimized MRM parameters of 34 amino acids and 3 ISs are summarized in Table 2.

### 2.3. Preparation of Calibration Standards and Quality Control Samples

Stock solutions (2.5 mg/mL) for each analyte were prepared separately and stored at −80°C. Gly, Ala, Ser, Val, Thr, Leu, Ile, Lys, Phe, Arg, Pro, Met, His, Cit, Sar, Apa, Amp, Aba, Opr, Hpr, Orn, Hia, and Kyn were dissolved in 5% methanol aqueous solutions, and Gln, Glu, and Trp were prepared in 0.2% FA aqueous solutions. Asp, Tyr, Asn, Cys, Cyss, Ahd, ADMA, and SDMA were in 4% HCl aqueous solutions. Stock solutions were further diluted with 5% methanol aqueous solution to obtain the following 4 groups of working solutions. Group A included Ala, Val, Thr, Leu, Ile, Lys, Glu, Phe, Arg, and Tyr (250 *μ*g/mL for every analyte). Group B included Gly, Ser, Asp, and Gln (250 *μ*g/mL for every analyte) and Pro, Asn, Met, and Trp (125 *μ*g/mL for every analyte). Group C included Cys, His, Cit, ADMA, and Cyss (250 *μ*g/mL for every analyte). Group D included Sar, Apa, Amp, Aba, Opr, Hpr, Orn, Ahd, Hia, SDMA, and Kyn (125 *μ*g/mL for each analyte).

The highest calibration standard solution was prepared by adding appropriate volumes of working solutions group A∼D into PBS (1x, namely, 0.01 mol/L) using previously reported methods [33–35]. Then, the other 8 calibration standard solutions were obtained by diluting the highest calibration standard solution with PBS. The final concentrations of calibration standard solutions were 1000, 2000, 4000, 8000, 10000, 20000, 40000, 60000, and 80000 ng/mL for Gly, Ala, Ser, Val, Thr, Leu, Ile, Asp, Lys, Gln, Glu, Phe, Arg, and Tyr; 500, 1000, 2000, 4000, 5000, 10000, 20000, 30000, and 40000 ng/mL for Pro, Asn, Met, and Trp; 100, 200, 400, 800, 1000, 2000, 4000, 6000, and 8000 ng/mL for Cys, His, Cit, ADMA, and Cyss; and 50, 100, 200, 400, 500, 1000, 2000, 3000, and 4000 ng/mL for Sar, Apa, Amp, Aba, Opr, Hpr, Orn, Ahd, Hia, SDMA, and Kyn, respectively.

Quality control (QC) samples were also separately prepared in the same way and at low, medium, and high concentrations (QC1∼3). The low, medium, and high concentrations of the QC samples were 2000, 10000, and 60000 ng/mL for Gly, Ala, Ser, Val, Thr, Leu, Ile, Asp, Lys, Gln, Glu, Phe, Arg, and Tyr; 1000, 5000, and 30000 ng/mL for Pro, Asn, Met, and Trp; 200, 1000, and 6000 ng/mL for Cys, His, Cit, ADMA, and Cyss; and 100, 500, and 3000 ng/mL for Sar, Apa, Amp, Aba, Opr, Hpr, Orn, Ahd, Hia, SDMA, and Kyn, respectively. All solutions were stored at −20°C.

### 2.4. Sample Pretreatment

Each tissue sample with a mass of about 100 mg was precisely weighed and added with a 5-fold mass of 0.9% saline before being homogenized by a superfine homogenizer at 15000 r/min for 2 min in the ice water bath to obtain tissue homogenate, and then the mixture was centrifuged for 15 min at 19060 ×g at 4°C after five minutes of ultrasonic treatment in the ice water bath. Then, a 50 *μ*L aliquot of the supernatant was transferred to a microcentrifuge tube, and 150 *μ*L of 0.2% FA acetonitrile solution (containing 400 ng/mL ISs) was added. The mixture was centrifuged again under the same conditions after being rested for 3 min and vortex-mixed for 2 min, and 2 *μ*L of supernatant was injected into the UHPLC-MS/MS system for analysis.

### 2.5. Method Validation

Method validation was performed according to Chinese Pharmacopoeia [36] and US Food and Drug Administration (FDA) guidance [37] and with reference to our previous report [38].

The selectivity was evaluated by comparing six different batches of the blank matrix to the corresponding spiked samples, and the responses of interferents in the blank matrix less than 20% of the low limit of quantitation (LLOQ) samples and 5% of ISs were considered acceptable.

The calibration standards were prepared in triplicates and measured three times on different days (at least 2 days). The calibration curve was regressed from the IS-adjusted peak area versus the nominal concentration under a 1/*X*^2^ weighting factor. LLOQ was defined as the lowest concentration point of the calibration curve. A deviation of backcalculation for each calibration standard within ±15% was thought to be acceptable, and for LLOQ, the deviation should be within ±20%.

The recovery and matrix effect were assessed by preparing six replicates of the QC sample at low and high concentration levels. The matrix effect was the ratio of the peak area in the spiked postextraction samples to solvent-substituted samples at the same concentration, and the recovery was the ratio of the peak area in the spiked samples to spiked postextraction samples at the same concentration.

The intra- and interday accuracy and precision were assessed using the QC samples at LLOQ, low, medium, and high concentration levels (*n* = 5). Samples were analyzed in three analytical lots on separate days (at least 2 days), and the relative standard deviation (RSD) % for inter- and intraday precision not more than 15% was regarded as acceptable (for LLOQ, not more than 20%). For intra- and interday accuracy, the relative error (RE) % within ±15% (for LLOQ, within ±20%) was considered reasonable.

The stability of each analyte was assessed at three concentration levels (low, medium, and high) using the QC samples (*n* = 3) under four different conditions: room temperature stability was evaluated after exposing samples at room temperature for 6 h; three freeze-thaw cycles stability was evaluated after freeze and thaw of samples from −20°C to room temperature three times; short-term stability was assessed by analyzing samples kept in the autosampler (4°C) for 24 h; and long-term stability was evaluated after the samples were stored at −20°C for 90 days.

The dilution effect of all the analytes was assessed by diluting the sample with a blank matrix into the calibration range and comparing the measured concentrations to the nominal concentrations. Each dilution factor should be assessed at least five times, and the RSD% and RE% should be less than 15% and within ±15%, respectively.

### 2.6. Study Population and Sample Collection

The experimental protocol was reviewed and approved by the Ethical Committee of Changzheng Hospital prior to specimen collection, and it was conducted in accordance with the Helsinki Declaration of 1964, as revised in 2013, and according to regulatory guidance. Informed consent was obtained from all participants enrolled in this study.

Between July 2016 and December 2017, 94 patients (male 56, female 38) with CRC were enrolled from Changzheng Hospital. None of the patients received neoadjuvant treatment. The median age of these patients was 60 (ranging from 32 to 87). 10 patients were diagnosed with stage I CRC, 33 patients with stage II, 45 patients with stage III, and 6 patients with stage IV. The demographic and clinical chemistry characteristics of these CRC patients are shown in Table 3.

The sample set including cancerous, paracancerous, and normal tissue samples was collected from each of these CRC patients and was named Tc, Tp, and Tn, respectively. All samples were immediately washed using 0.9% icy saline solution, and the surfaces were subsequently dried by the filter tissue. The samples were stored at −80°C within cryotubes until analysis.

### 2.7. Data Analysis

Data were analyzed statistically, and graphs were generated by GraphPad Prism 6.01 for Windows (GraphPad Software, Inc., La Jolla, CA, USA). A nonparametric test (Friedman test) was performed to compare the content differences of 34 amino acids between sample sets. The *p* value less than 0.05 was considered statistically significant.

## 3. Results and Discussion

### 3.1. Method Development

Many studies have published the quantitative analyses of amino acids in plasma [23, 24, 28, 31, 32], but these methods have never been applied in tissue homogenate. In this study, we developed a robust method for the quantitative analysis of underivatized amino acids in human tissue by UHPLC-MS/MS.

**Figure 2:**
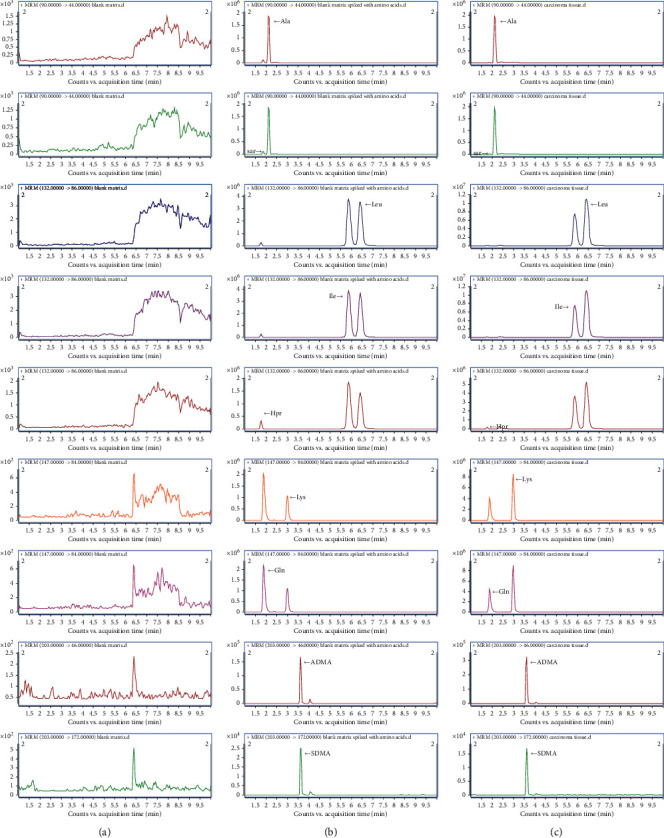
The representative MRM chromatograms of Ala, Sar, Leu, Ile, Hpr, Lys, Gln, ADMA, and SDMA: (a) blank matrix; (b) blank matrix spiked with 34 amino acid and 3 ISs; (c) cancerous tissue sample.

As for optimization of ESI-MS/MS conditions, this study highlighted the importance of quantifying the isomeric analytes using two strategies. For compounds that shared the same MRM transitions, such as Ala and Sar, modifications of the mobile phase and its gradient, as well as the column optimization, were tried to make sure they were completely separated in chromatography. The coelution of the other two pairs of standards, such as Leu, Ile, and Hpr and Lys and Gln, was also avoided at the same time. If the isomeric analytes had a similar retention time, another MRM transition was chosen to separate them on different mass spectrometer channels. For example, the MRM transition for ADMA was set at 203/46 instead of 203/70 and 203/172 for SDMA. The representative MRM chromatograms of Ala, Sar, Leu, Ile, Hpr, Lys, Gln, ADMA, and SDMA are shown in Figure 2.

During the selection of ISs, the ideal condition was an isotope-labeled internal standard for each analyte, but the major problem was the high expense and longer delivery time. Besides, those amino acids were of the same structure, so it was acceptable to use a structural analog as the internal standard for analytes. Furthermore, method verification in our experiment was currently acceptable as specified by Chinese Pharmacopoeia and FDA guidelines. Hence, the practical value was the availability of three isotopically labeled amino acids, which could facilitate application while making research less expensive.

In terms of optimization of chromatographic conditions, generally, analysis using sub-2 *μ*m columns yielded a greater (S/N) due to the reduction in band broadening and thus an increase in sensitivity. We actually conducted the analysis by using reverse-phase LC columns with 1.8 and 5.0 *μ*m packing materials, which generated a similar chromatographic peak resolution. Moreover, ion suppression from the coeluting peak was alleviated when the 5.0 *μ*m column was used. Since gradual accumulation of small amounts of protein and/or particulates might occur and become noticeable after injection of a large number of samples owing to the incomplete efficiency of protein removal (only about 95%∼99%) [39], a wash step of the column was set between different analysis batches. Besides, the use of a column of 5 *μ*m particle size was considered to be less vulnerable pollution than one of 1.8 *μ*m particle size. Based on our previous reports [38], the adding of HFBA could lead to the best performance separation for amino acids. Therefore, we found that 0.02% HFBA and 0.2% FA aqueous solution with methanol could result in better separation of compounds and chromatographic peaks shapes and higher signal response (S/N) for most analytes. Also, great abundance and high ionization efficiency were obtained in the positive ionization mode for all the analytes.

The selection of an appropriate matrix for calibration samples and the QC samples preparation was an important part of methodological development when LC-MS was used for the quantitative analysis of endogenous compounds in biological samples. There were two main approaches to this problem: the first was to dissolve alternative analytes in the real matrix and the other was to use real analytes in an alternative matrix [35]. The ideal substitutive matrix should be completely analyte-free and identical to the real matrix in terms of analyte solubility and extractability, but it was unpractical for the detection of endogenous compounds. In our experiment, plasma was processed using neutral decolorizing carbon for stripping some endogenous carbohydrates [40]. This approach was effective for carbohydrates but not for all amino acids because the prepared plasma still contained a high concentration of amino acids. Therefore, the calibration and the QC samples could be prepared in an artificial matrix only as it was impossible to make an “analyte-free” matrix. As human biofluid usually contains a variety of proteins, fatty acids, and electrolytes, which is hard for simulation, some research studies documented that the PBS [33, 34] or mobile phase [41] could be treated as the matrix when the calibration and the QC samples were prepared. In addition, the pH value, osmotic pressure, and ion concentration of PBS were closer to those of the biofluid of humans than the mobile phase [35]. As such, PBS was applied as the “mimic tissue fluid” to prepare both the calibration and the QC samples.

When it came to optimization of sample preparation, treatment of the tissue homogenate commonly involved protein precipitation (PPT), liquid-liquid extraction, and solid phase extraction. The PPT method was considered the best method in that it was user-friendly, inexpensive, and suitable for high-throughput biological sample pretreatment. It was also validated in our previous study for the assay of 18 plasma amino acids by a UHPLC-MS/MS method [38]. By adding a 3-fold volume of precipitator (0.2% FA acetonitrile solution containing each IS of 400 ng/mL), stable and optimal recovery as well as matrix effect was achieved, so this pretreatment method was applied in this improved method.

In all, the quantification of amino acids in human plasma by both GC-MS- and LC-MS-based mass spectroscopy was well established [23, 24, 28, 31, 32] and would ideally suit our validation study after improvement. As the key differences for LC-MS were often in terms of sample preparation and separation parameters, the comparison of those published LC-MS methods with the UHPLC-MS/MS method established by us could find that (a) our analysis was time-saving and economical and without any derivatization process; (b) a unique chromatographic configuration minimized ion suppression and yielded excellent analytic performance, especially for the isomers; and (c) this method could easily be extended to different sample matrices, such as plasma (data not shown).

### 3.2. Method Validation Results

#### 3.2.1. Specificity

The representative total ion current chromatograms and MRM chromatograms of blank sample, blank sample spiked with 34 amino acids and 3 ISs, and real CRC samples are shown in Supplementary Material Figure S1. The retention time of the 34 amino acids is shown in Table 4. No interfering peaks from endogenous matrix substances were shown at the retention time of 34 amino acids and 3 ISs, suggesting satisfactory separation and selectivity.

#### 3.2.2. Linearity of Calibration Curves and LLOQ

The linear equations were regressed to calculate the measured concentrations in all samples within the analytical runs. Good correlation coefficients (*r* > 0.99) were observed for all analytes in their corresponding calibration ranges. The regression equations, coefficients, calibration ranges, and LLOQ for the 34 amino acids and ISs are shown in Table 4.

#### 3.2.3. Recovery and Matrix Effect

The average recovery results of the 34 amino acids and 3 ISs using QC1 and QC3 samples ranged from 39.00% to 146.95% (RSD 0.44%∼7.40%). The average matrix effect results using the same two samples ranged from 49.45% to 173.63% (RSD 0.61%∼12.97%), indicating that the extraction procedure was consistent and stable (Supplementary Material Table S2).

#### 3.2.4. Inter- and Intraday Accuracy and Precision

The intra- and interday accuracy and precision of this method were assessed using the LLOQ, QC1, QC2, and QC3 samples. The deviations (RE%) of intraday ranged from −13.52% to 14.21%, and the RSD were less than 8.57%, while the deviations of interday ranged from −14.52% to 12.59%, and the RSD was not more than 10.31%. The intra- and interday accuracy of the LLOQ sample ranged from −18.03% to 16.99%, and precision was less than 13.25%. The results are shown in Supplementary Material Table S3.

#### 3.2.5. Stability

The stability results of different conditions are shown in Supplementary Material Table S4. It demonstrated that all the analytes were stable with the accuracy within ±15% under different conditions.

#### 3.2.6. Dilution Effect

The dilution effect results showed that the accuracy and precision for 8-time dilution were acceptable (RE ranged from −12.86% to 14.42%, RSD ≤5.46% for all the analytes). The results are shown in Supplementary Material Table S5.

### 3.3. Application in Determination of Clinical Samples

**Figure 3:**
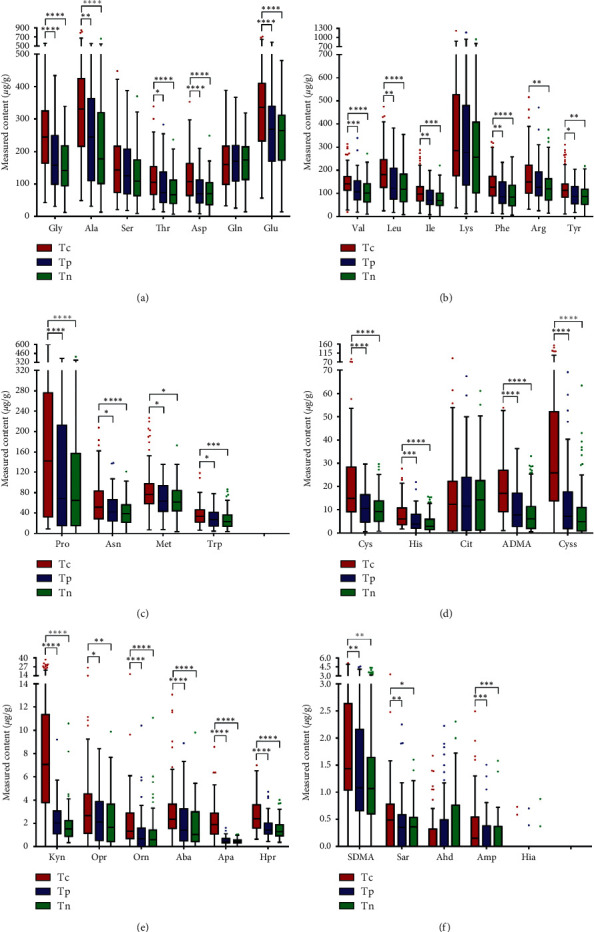
The contents of 34 amino acids in cancerous, paracancerous, and normal tissue from 94 CRC patients (^*∗*^*p* < 0.05, ^*∗∗*^*p* < 0.01, ^*∗∗∗*^*p* < 0.001, and ^*∗∗∗∗*^*p* < 0.0001).

This targeted UHPLC-MS/MS method was successfully applied to simultaneously determine 34 amino acids in tumor tissue samples.  Figure 3 and Table 5 depict the contents, median, and SD values of 34 amino acids in cancerous, paracancerous, and normal tissue from 94 CRC patients. As expected, the contents of some amino acids were significantly different between sample types. For example, Gly in Tc sample was markedly higher than that in Tp and Tn samples (each *p* < 0.0001). In addition, significantly increased contents were also found in Tc sample for the Asp, Glu, Pro, Cys, ADMA, Cyss, Kyn, Orn, Aba, Apa, and Hpr (each *p* < 0.0001). This supported the trait of altered metabolism of cancer, and it was encouraging that the candidate metabolites varied so much between cancer and normal tissue. However, it was not enough to diagnose the CRC by the amino acid profiles alone because the samples in our study were limited, and further research was needed to evaluate their potential as biomarkers for CRC diagnosis and treatment.

## 4. Conclusion

A simple, rapid, sensitive, and efficient targeted UHPLC-MS/MS method was developed for the determination of 34 amino acids with analytical time less than 10 min in tumor tissues, which was validated for selectivity, linearity, extraction recovery, matrix effect, intra- and interday accuracy and precision, and stability. The use of diluted PBS as the “mimic tissue fluid” could prevent serious interference from endogenous amino acids, which was proved to be simple and efficient. Enough retention was achieved for the highly polar amino acid analytes in the C_18_ column by using the HFBA, an ion-pairing reagent, as the mobile phase addictive, without any derivatization procedure. The one step PPT method for 100 mg cancer tissue supported the high-throughput testing. In summary, this UHPLC-MS/MS method was successfully applied to plot the profiles of 34 amino acids in cancerous, paracancerous, and normal tissue from CRC patients, which may be of help for the diagnosis and treatment of CRC in the future.

## Figures and Tables

**Table 1:** The summarized variations of amino acids in blood, urine, feces, and cancerous tissue of CRC patients.

Amino acids	Variations				References
	In blood^*∗*^	In urine^*∗*^	In feces^*∗*^	In tissue^#^	
Gly	↓			↑	[1, 2]
Ala		↑		↑	[3–5]
Ser	↑	↓			[3, 6, 7]
Val	↓	↑			[2, 6, 7]
Thr	↓				[2]
Leu	↓↑	↑			[3, 5–7]
Ile		↑			[6–8]
Asp	↓↑	↓		↑	[3, 4, 6, 7, 9]
Gln	↓	↑			[3, 8]
Glu	↑			↑	[4, 8, 9]
Phe	↓			↑	[3, 10]
Arg		↑			[6, 7]
Tyr		↑		↑	[3, 10]
Pro	↓		↑	↑	[3, 11, 12]
Met	↓	↓			[5–7]
Trp		↑			[3]
Cys			↑	↑	[4, 11]
His	↓	↓			[3, 6, 7]
Cit	↑				[3]
ADMA	↑				[13–15]
Cyss	↑				[9]
Aba				↑	[16, 17]
Opr				↑	[4]
Hpr	↓				[3]
Orn	↑				[3]
Ahd	↓				[5]
Hia	↑	↓			[5, 18]
SDMA	↑				[14, 15]
Kyn	↑	↓			[9, 18]

*Note*. ^*∗*^CRC patients vs. healthy volunteers; ↑: increase; ↓: decrease; ↓↑: increase and decrease. ^#^The cancerous tissue of CRC patients vs. the normal tissue of CRC patients.

**Table 2:** The optimized MRM parameters of 34 amino acids and 3 ISs (ESI positive).

Analyte	Molecular weight	Precursor ion	Product ion	Fragmentor (V)	Collision energy (eV)
Gly	75.07	76	30	50	13
Ala	89.09	90	44	50	8
Ser	105.09	106	60	65	9
Val	117.15	118	72	60	7
Thr	119.12	120	74	65	9
Leu	131.17	132	86	65	7
Ile	131.17	132	86	65	7
Asp	133.10	134	74	65	12
Lys	146.19	147	84	70	11
Gln	146.14	147	84	65	12
Glu	147.13	148	84	70	12
Phe	165.19	166	120	65	10
Arg	174.20	175	70	90	16
Tyr	181.19	182	136	70	11
Pro	115.13	116	70	70	15
Asn	132.12	133	74	60	11
Met	149.21	150	56	65	13
Trp	204.23	205	188	70	5
Cys	121.16	122	59	60	21
His	155.15	156	110	80	13
Cit	175.19	176	159	70	7
ADMA	202.25	203	46	90	16
Cyss	240.30	241	152	80	12
Sar	89.09	90	44	55	10
Apa	89.09	90	30	60	12
Amp	103.12	104	30	60	11
Aba	103.12	104	87	65	9
Opr	129.11	130	84	75	12
Hpr	131.13	132	86	75	13
Orn	132.16	133	70	65	10
Ahd	161.16	162	98	65	11
Hia	179.17	180	105	65	6
SDMA	202.25	203	172	90	14
Kyn	208.21	209	192	70	7
Ala-d4	93.12	94	48	50	9
Met-d3	152.23	153	56	65	13
Phe-d5	170.22	171	125	70	11

**Table 3:** The demographic and clinical chemistry characteristics of CRC patients.

Items	Total	Male	Female
Number of patients	94	56	38
Age (median, range)	60, (32∼87)	58, (32∼87)	61, (38∼80)
Number of patients with TNM stage			
Stage I	10	3	7
Stage II	33	18	15
Stage III	45	33	12
Stage IV	6	2	4

**Table 4:** The retention times, regression equations, coefficients, calibration ranges, and LLOQ for 34 amino acids and each corresponding IS.

Analyte	Retention time (min)	Regression equation (*n* = 9)	Coefficient *R*^2^	Calibration range (ng/ml)	LLOQ (ng/ml)	IS
Gly	1.866	*y* = 1.973405 × 10^−5^*x* + 0.013171	0.99434	1000∼80000	1000	Phe-d5
Ala	2.118	*y* = 0.002292*x* − 0.471134	0.99538	1000∼80000	1000	Ala-d4
Ser	1.809	*y* = 1.120434 × 10^−4^*x* + 0.405218	0.99220	1000∼80000	1000	Phe-d5
Val	3.607	*y* = 0.003533*x* − 0.598212	0.99472	1000∼80000	1000	Ala-d4
Thr	2.026	*y* = 0.001422*x* + 0.129849	0.99604	1000∼80000	1000	Met-d3
Leu	6.402	*y* = 0.021977*x* − 7.784039	0.99375	1000∼80000	1000	Met-d3
Ile	5.863	*y* = 0.022788*x* − 6.270995	0.99535	1000∼80000	1000	Met-d3
Asp	1.787	*y* = 4.507537 × 10^−4^*x* + 0.522395	0.98923	1000∼80000	1000	Met-d3
Lys	2.994	*y* = 5.106616 × 10^−4^*x* + 0.021605	0.98157	1000∼80000	1000	Phe-d5
Gln	1.882	*y* = 0.005491*x* + 4.126505	0.99513	1000∼80000	1000	Met-d3
Glu	2.066	*y* = 0.001872*x* + 0.886773	0.99897	1000∼80000	1000	Met-d3
Phe	7.554	*y* = 0.006928*x* − 3.149161	0.99258	1000∼80000	1000	Phe-d5
Arg	3.329	*y* = 0.001577*x* + 0.298584	0.99370	1000∼80000	1000	Met-d3
Tyr	4.427	*y* = 1.241443 × 10^−4^*x* + 0.056822	0.99250	1000∼80000	1000	Phe-d5
Pro	2.146	*y* = 0.004142*x* + 0.052886	0.99646	500∼40000	500	Ala-d4
Asn	1.770	*y* = 9.372446 × 10^−4^*x* + 0.471997	0.99304	500∼40000	500	Met-d3
Met	3.636	*y* = 0.001404*x* − 0.179621	0.99310	500∼40000	500	Met-d3
Trp	8.755	*y* = 3.881832 × 10^−4^*x* − 0.034688	0.99746	500∼40000	500	Phe-d5
Cys	2.056	*y* = 6.151545 × 10^−4^*x* − 0.024106	0.99544	100∼8000	100	Met-d3
His	2.695	*y* = 0.005120*x* + 0.129674	0.98902	100∼8000	100	Met-d3
Cit	2.139	*y* = 0.00316*x* + 0.123514	0.99490	100∼8000	100	Met-d3
ADMA	3.610	*y* = 2.22466 × 10^−4^*x* − 0.008033	0.99537	100∼8000	100	Phe-d5
Cyss	1.843	*y* = 3.122902 × 10^−5^*x* + 0.007852	0.99611	100∼8000	100	Phe-d5
Sar	1.856	*y* = 7.269948 × 10^−4^*x* + 0.017312	0.99724	50∼4000	50	Phe-d5
Apa	2.432	*y* = 7.290745 × 10^−4^*x* − 0.009428	0.99649	50∼4000	50	Met-d3
Amp	3.181	*y* = 1.459612 × 10^−4^*x* + 8.722727 × 10^−4^	0.98729	50∼4000	50	Phe-d5
Aba	2.771	*y* = 0.005955*x* − 0.085380	0.99898	50∼4000	50	Met-d3
Opr	2.567	*y* = 1.048082 × 10^−4^*x* + 0.035973	0.99634	50∼4000	50	Phe-d5
Hpr	1.759	*y* = 0.017388*x* + 0.017864	0.99533	50∼4000	50	Met-d3
Orn	2.676	*y* = 0.001756*x* + 0.236473	0.99406	50∼4000	50	Met-d3
Ahd	2.577	*y* = 0.003022*x* − 0.039314	0.99902	50∼4000	50	Ala-d4
Hia	7.210	*y* = 0.001064*x* − 0.002872	0.99309	50∼4000	50	Phe-d5
SDMA	3.619	*y* = 7.281465 × 10^−5^*x* − 0.001614	0.99514	50∼4000	50	Phe-d5
Kyn	6.298	*y* = 3.698408 × 10^−4^*x* − 4.537424 × 10^−4^	0.99744	50∼4000	50	Phe-d5

**Table 5:** The median and SD values of 34 amino acids in cancerous, paracancerous, and normal tissue from 94 CRC patients.

Analyte	Cancerous tissue (*μ*g/g)		Paracancerous tissue (*μ*g/g)		Normal tissue (*μ*g/g)	
	Median	SD	Median	SD	Median	SD
Gly	244.454	110.611	156.906	91.526	141.401	79.605
Ala	330.693	163.616	244.684	145.235	177.389	137.482
Ser	143.072	92.541	124.721	84.070	109.057	78.118
Val	141.541	58.259	107.203	56.744	102.171	54.720
Thr	104.922	63.748	73.603	59.520	67.197	54.334
Leu	181.020	94.785	122.750	84.062	118.304	79.573
Ile	97.020	58.074	71.391	43.525	70.712	41.863
Asp	107.197	67.386	69.694	44.618	70.323	42.289
Lys	284.995	231.061	278.033	248.794	255.968	217.480
Gln	159.779	77.801	170.369	70.657	173.578	64.244
Glu	335.986	145.846	268.418	116.329	264.143	100.596
Phe	127.176	70.195	85.905	58.385	84.676	57.804
Arg	150.227	97.943	127.102	79.598	119.700	72.975
Tyr	113.370	60.918	88.310	47.617	87.389	46.861
Pro	141.950	152.552	68.731	110.943	65.552	99.701
Asn	54.833	42.362	42.362	28.168	39.203	25.920
Met	76.329	45.541	63.348	31.676	61.494	32.014
Trp	32.690	19.826	27.032	17.460	23.207	17.925
Cys	15.593	16.903	10.482	6.979	9.233	6.233
His	6.044	5.366	4.033	4.267	3.050	4.008
Cit	16.222	16.414	16.281	14.671	17.802	13.330
ADMA	17.082	11.799	7.841	9.571	6.117	8.310
Cyss	26.370	33.970	10.720	14.34	6.713	11.742
Sar	0.611	0.490	0.520	0.384	0.491	0.281
Apa	1.934	1.456	0.529	0.266	0.474	0.192
Amp	0.525	0.474	0.469	0.284	0.478	0.286
Aba	2.341	2.356	2.069	1.857	1.246	1.745
Opr	2.982	3.705	2.637	2.047	2.514	1.955
Hpr	2.390	1.385	1.425	0.831	1.282	0.791
Orn	1.551	2.266	1.043	1.634	1.101	1.735
Ahd	0.481	0.390	0.745	0.514	0.862	0.458
Hia	0.656	0.103	0.546	0.220	0.625	0.358
SDMA	1.525	1.093	1.116	1.139	1.145	1.017
Kyn	7.065	8.108	2.043	1.553	1.536	1.488

## Abbreviations

Aba: 4-Aminobutyric acid
ADMA: Asymmetric dimethylarginine
Ahd: 2‐Amino‐L‐hexanoic diacid
Ala: L-Alanine
Ala-d4: L-Alanine-d4
Amp: 3-Amino-2-methylpropanoic acid
Apa: 3-Aminopropanoic acid
Arg: L-Arginine
Asn: L-Asparagine
Asp: L-Aspartic acid
Cit: L-Citrulline
CRC: Colorectal cancer
Cys: L-Cysteine
Cyss: L-Cystine
ESI: Electrospray ionization
FA: Formic acid
FDA: Food and drug administration
GC-MS: Gas chromatography-mass spectrometry
Gln: L-Glutamine
Glu: L-Glutamic acid
Gly: Glycine
HCl: Hydrochloric acid
HFBA: Heptafluorobutyric acid
Hia: Hippuric acid
His: L-Histidine
Hpr: 4-Hydroxy-L-proline
HPLC: High-performance liquid chromatography
Ile: L-Isoleucine
IS: Internal standard
Kyn: L-Kynurenine
LC-MS: Liquid chromatography-mass spectrometry
Leu: L-Leucine
LLOQ: Low limit of quantitation
Lys: L-Lysine
Met: L-Methionine
Met-d3: L-Methionine-d3
MRM: Multiple reaction monitoring
Opr: 5-Oxo-L-proline
Orn: L-Ornithine
PBS: Phosphate-buffered solution
Phe: L-Phenylalanine
Phe-d5: L-Phenylalanine-d5
PPT: Protein precipitation
Pro: L-Proline
QC: Quality control
RE: Relative error
RSD: Relative standard deviation
Sar: Sarcosine
SDMA: Symmetric dimethylarginine
Ser: L-Serine
TCA: Tricarboxylic acid
Thr: L-Threonine
Trp: L-Tryptophan
Tyr: L-Tyrosine
UHPLC-MS/MS: Ultrahigh-performance liquid chromatography-tandem mass spectrometry
Val: L-Valine.

## Supplementary Materials

Supplementary Materials TABLE S1: the control substances of 34 amino acids and 3 ISs. TABLE S2: the extraction recovery and matrix effect of 34 amino acids and 3 ISs (*n* = 6). TABLE S3: the intra- and interday accuracy and precision of 34 amino acids (*n* = 5). TABLE S4: the stability results of 34 amino acids (*n* = 3). TABLE S5: the dilution effect results of 34 amino acids (*n* = 3). FIGURE S1: the representative total ion current chromatograms and MRM chromatograms of 34 amino acids and 3 ISs—(a) blank matrix; (b) blank matrix spiked with 34 amino acid and 3 ISs; (c) cancerous tissue sample.
