# Supplementary material for: Simultaneous Determination of 34 Amino Acids in Tumor Tissues from Colorectal Cancer Patients Based on the Targeted UHPLC-MS/MS Method
Source: J Anal Methods Chem. 2020 Aug 1;2020:4641709. doi: 10.1155/2020/4641709 (PMC7416278; doi:10.1155/2020/4641709)
Supplement: Supplementary Materials — TABLE S1: the control substances of 34 amino acids and 3 ISs. TABLE S2: the extraction recovery and matrix effect of 34 amino acids and 3 ISs (n = 6). TABLE S3: the intra- and interday accuracy and precision of 34 amino acids (n = 5). TABLE S4: the stability results of 34 amino acids (n = 3). TABLE S5: the dilution effect results of 34 amino acids (n = 3). FIGURE S1: the representative total ion current chromatograms and MRM chromatograms of 34 amino acids and 3 ISs—(a) blank matrix; (b) blank matrix spiked with 34 amino acid and 3 ISs; (c) cancerous tissue sample. [file 4641709.f1.docx]

**Supplementary materials**

**Simultaneous determination of 34 amino acids in tumor tissues from colorectal cancer patients based on targeted UHPLC-MS/MS method**

Yang Yang,^1,2,3^ · Feng Zhang,^1^ · Shouhong Gao,^1^ · Zhipeng Wang,^1^ · Mingming Li,^1^ · Hua Wei,^1^ · Renqian Zhong,^3,*^ and Wansheng Chen^1,*^

*^1^ Department of Pharmacy, Changzheng Hospital, the Second Military Medical University of CPLA, Shanghai 200003, PR. China*

*^2^ Department of Pharmacy, the 71st Group Army Hospital of CPLA Army / the affiliated Huaihai Hospital of Xuzhou Medical University, Xuzhou 221004, PR. China*

*^3^ Department of Laboratory Diagnostics, Changzheng Hospital, the Second Military Medical University of CPLA, Shanghai 200003, PR. China*

Correspondence should be addressed to Renqian Zhong; zhongrq@smmu.edu.cn and Wansheng Chen; chenwansheng@smmu.edu.cn

**TABLE S1: The control substances of 34 amino acids and 3 ISs.**

| **Control substance** | **Molecular  weight** | **Production company** | **Batch number** | **Purity** | **Structure formula** |
| --- | --- | --- | --- | --- | --- |
| Gly | 75.07 | National Institutes for food and drug control of China | 140624-201506 | 99.9% |  |
| Ala | 89.09 | National Institutes for food and drug control of China | 140624-201506 | 99.9% |  |
| Ser | 105.09 | National Institutes for food and drug control of China | 140624-201506 | 99.9% |  |
| Val | 117.15 | National Institutes for food and drug control of China | 140624-201506 | 99.9% |  |
| Thr | 119.12 | National Institutes for food and drug control of China | 140624-201506 | 99.9% |  |
| Leu | 131.17 | National Institutes for food and drug control of China | 140624-201506 | 99.9% |  |
| Ile | 131.17 | National Institutes for food and drug control of China | 140624-201506 | 99.9% |  |
| Asn | 132.12 | Shanghai Yuanye biotech Co., Ltd. (China) | SM0503GA13 | ≥98% |  |
| Asp | 133.10 | National Institutes for food and drug control of China | 140624-201506 | 99.9% |  |
| Lys ·HCl | 182.65 | National Institutes for food and drug control of China | 140624-201506 | 100% | ·HCl |
| Gln | 146.14 | Dalian Meilun biotech Co., Ltd. (China) | J1105AS | >98% |  |
| Glu | 147.13 | National Institutes for food and drug control of China | 140624-201506 | 99.9% |  |
| Met | 149.21 | National Institutes for food and drug control of China | 140624-201506 | 99.9% |  |
| Cys | 121.16 | Shanghai Yuanye biotech Co., Ltd. (China) | SA0410GA14 | ≥98% |  |
| Cyss | 240.30 | National Institutes for food and drug control of China | 140624-201506 | 99.8% |  |
| Arg | 174.20 | National Institutes for food and drug control of China | 140624-201506 | 99.9% |  |
| Orn ·HCl | 168.62 | Dalian Meilun biotech Co., Ltd. (China) | A0821AS | >98% | ·HCl |
| Cit | 175.19 | Dalian Meilun biotech Co., Ltd. (China) | M0510A | >99% |  |
| ADMA ·2HCl | 275.18 | Sigma-aldrich LLC.  (Darmstadt, Germany) | SLBK4505V | ≥98% | ·2HCl |
| SDMA | 202.25 | Dalian Meilun biotech Co., Ltd. (China) | 20130525 | >95% |  |
| Pro | 115.13 | National Institutes for food and drug control of China | 140624-201506 | 99.9% |  |
| Opr | 129.11 | Dalian Meilun biotech Co., Ltd. (China) | M0108A | >99% |  |
| Hpr | 131.13 | Dalian Meilun biotech Co., Ltd. (China) | N0524AS | >98% |  |
| His | 155.15 | National Institutes for food and drug control of China | 140624-201506 | 99.9% |  |
| Phe | 165.19 | National Institutes for food and drug control of China | 140624-201506 | 100% |  |
| Tyr | 181.19 | National Institutes for food and drug control of China | 140624-201506 | 99.8 |  |
| Trp | 204.23 | National Institutes for food and drug control of China | 140624-201506 | 99.9 |  |
| Kyn | 208.21 | Dalian Meilun biotech Co., Ltd. (China) | M0208AS | >98% |  |
| Sar | 89.09 | Dalian Meilun biotech Co., Ltd. (China) | A0810A | >99% |  |
| Hia | 179.17 | Dalian Meilun biotech Co., Ltd. (China) | 20130510 | >98% |  |
| Apa | 89.09 | Shanghai Yuanye biotech Co., Ltd. (China) | G31A4G1 | ≥98% |  |
| Amp | 103.12 | Shanghai Yuanye biotech Co., Ltd. (China) | A21D7L27358 | ≥98% |  |
| Aba | 103.12 | Dalian Meilun biotech Co., Ltd. (China) | A0612AS | >98% |  |
| Ahd | 161.16 | Sigma-aldrich LLC.  (Darmstadt, Germany) | BCBK5036V | ≥98% |  |
| Ala-d4 | 93.12 | Toronto Research Chemicals Inc. (Toronto, Canada) | 11-MIC-77-1 | - |  |
| Met-d3 | 152.23 | Toronto Research Chemicals Inc. (Toronto, Canada) | 22-SSR-125-1 | - |  |
| Phe-d5 | 170.22 | Toronto Research Chemicals Inc. (Toronto, Canada) | 23-XJZ-28-1 | - |  |

**TABLE S2: The extraction recovery and matrix effect of 34 amino acids and 3 ISs (n=6).**

| **Analyte** | **Concentration (ng/ml)** |  | **Extraction recovery** | |  | **Matrix effect** | |
| --- | --- | --- | --- | --- | --- | --- | --- |
|  |  |  | **Mean±SD (%)** | **RSD (%)** |  | **Mean±SD (%)** | **RSD (%)** |
| Gly | 2000 |  | 78.41**±**1.73 | 2.21 |  | 107.34**±**3.26 | 3.03 |
|  | 60000 |  | 79.85**±**2.34 | 2.93 |  | 105.93**±**3.88 | 3.66 |
| Ala | 2000 |  | 65.30**±**1.33 | 2.04 |  | 109.72**±**1.76 | 1.61 |
|  | 60000 |  | 74.12**±**1.84 | 2.49 |  | 126.52**±**2.83 | 2.24 |
| Ser | 2000 |  | 88.58**±**0.96 | 1.09 |  | 108.89**±**2.05 | 1.88 |
|  | 60000 |  | 72.62**±**2.80 | 3.85 |  | 114.95**±**3.93 | 3.42 |
| Val | 2000 |  | 72.24**±**0.99 | 1.36 |  | 116.81**±**3.33 | 2.85 |
|  | 60000 |  | 100.04**±**1.59 | 1.59 |  | 94.35**±**2.72 | 2.88 |
| Thr | 2000 |  | 71.90**±**0.76 | 1.06 |  | 110.79**±**1.63 | 1.47 |
|  | 60000 |  | 80.94**±**2.91 | 3.59 |  | 115.09**±**2.85 | 2.48 |
| Leu | 2000 |  | 89.56**±**0.70 | 0.78 |  | 106.99**±**1.03 | 0.96 |
|  | 60000 |  | 105.05**±**1.83 | 1.74 |  | 98.26**±**0.95 | 0.97 |
| Ile | 2000 |  | 89.07**±**0.39 | 0.44 |  | 106.32**±**1.30 | 1.22 |
|  | 60000 |  | 103.80**±**1.42 | 1.37 |  | 99.65**±**0.61 | 0.61 |
| Asp | 2000 |  | 96.07**±**0.61 | 0.64 |  | 102.75**±**1.71 | 1.66 |
|  | 60000 |  | 80.10**±**1.99 | 2.49 |  | 112.66**±**2.60 | 2.31 |
| Lys | 2000 |  | 44.33**±**1.57 | 3.55 |  | 125.43**±**3.14 | 2.50 |
|  | 60000 |  | 47.09**±**1.47 | 3.11 |  | 119.72**±**5.16 | 4.31 |
| Gln | 2000 |  | 74.16**±**0.75 | 1.02 |  | 108.31**±**1.28 | 1.18 |
|  | 60000 |  | 80.52**±**2.31 | 2.86 |  | 107.98**±**1.91 | 1.77 |
| Glu | 2000 |  | 75.54**±**0.56 | 0.75 |  | 114.98**±**1.60 | 1.39 |
|  | 60000 |  | 78.74**±**1.75 | 2.22 |  | 120.36**±**2.70 | 2.24 |
| Phe | 2000 |  | 84.86**±**0.69 | 0.81 |  | 109.43**±**1.10 | 1.01 |
|  | 60000 |  | 102.20**±**0.97 | 0.95 |  | 99.86**±**0.63 | 0.63 |
| Arg | 2000 |  | 54.60**±**0.82 | 1.49 |  | 119.59**±**2.07 | 1.73 |
|  | 60000 |  | 57.64**±**1.76 | 3.06 |  | 120.44**±**5.21 | 4.33 |
| Tyr | 2000 |  | 82.21**±**1.80 | 2.19 |  | 115.87**±**1.91 | 1.65 |
|  | 60000 |  | 85.29**±**2.07 | 2.43 |  | 120.34**±**1.92 | 1.60 |
| Pro | 1000 |  | 146.95**±**2.65 | 1.81 |  | 49.45**±**3.00 | 6.06 |
|  | 30000 |  | 125.79**±**2.49 | 1.98 |  | 71.42**±**9.27 | 12.97 |
| Asn | 1000 |  | 72.30**±**1.10 | 1.52 |  | 107.21**±**1.27 | 1.19 |
|  | 30000 |  | 78.01**±**2.28 | 2.92 |  | 110.27**±**4.07 | 3.69 |
| Met | 1000 |  | 57.55**±**1.37 | 2.38 |  | 161.85**±**6.53 | 4.04 |
|  | 30000 |  | 73.44**±**2.21 | 3.00 |  | 157.26**±**0.96 | 0.61 |
| Trp | 1000 |  | 91.05**±**0.80 | 0.88 |  | 113.74**±**3.16 | 2.78 |
|  | 30000 |  | 94.30**±**1.60 | 1.70 |  | 113.28**±**1.25 | 1.11 |
| Cys | 200 |  | 49.73**±**2.82 | 5.67 |  | 129.29**±**4.44 | 3.44 |
|  | 6000 |  | 85.53**±**3.33 | 3.90 |  | 119.87**±**1.35 | 1.13 |
| His | 200 |  | 42.61**±**1.47 | 3.44 |  | 129.10**±**4.10 | 3.17 |
|  | 6000 |  | 65.74**±**1.55 | 2.35 |  | 111.82**±**3.03 | 2.71 |
| Cit | 200 |  | 59.71**±**2.32 | 3.89 |  | 135.83**±**5.27 | 3.88 |
|  | 6000 |  | 68.43**±**5.06 | 7.40 |  | 125.26**±**2.66 | 2.12 |
| ADMA | 200 |  | 39.00**±**2.62 | 6.71 |  | 173.63**±**8.27 | 4.76 |
|  | 6000 |  | 71.51**±**2.07 | 2.90 |  | 114.45**±**3.73 | 3.26 |
| Cyss | 200 |  | 63.40**±**4.50 | 7.10 |  | 104.02**±**4.54 | 4.36 |
|  | 6000 |  | 54.63**±**1.88 | 3.44 |  | 112.23**±**4.64 | 4.14 |
| Sar | 100 |  | 75.54**±**1.22 | 1.62 |  | 109.39**±**2.88 | 2.63 |
|  | 3000 |  | 84.08**±**2.45 | 2.91 |  | 111.77**±**4.05 | 3.63 |
| Apa | 100 |  | 74.66**±**2.61 | 3.50 |  | 105.39**±**2.92 | 2.77 |
|  | 3000 |  | 79.42**±**3.05 | 3.83 |  | 107.99**±**3.50 | 3.24 |
| Amp | 100 |  | 75.12**±**1.13 | 1.50 |  | 111.20**±**3.03 | 2.73 |
|  | 3000 |  | 85.31**±**2.78 | 3.26 |  | 107.99**±**2.22 | 2.06 |
| Aba | 100 |  | 74.62**±**0.63 | 0.85 |  | 109.71**±**1.97 | 1.79 |
|  | 3000 |  | 86.86**±**2.56 | 2.94 |  | 107.87**±**2.52 | 2.34 |
| Opr | 100 |  | 86.96**±**5.36 | 6.17 |  | 130.15**±**8.08 | 6.21 |
|  | 3000 |  | 84.91**±**3.63 | 4.27 |  | 127.44**±**2.96 | 2.32 |
| Hpr | 100 |  | 57.83**±**1.42 | 2.45 |  | 110.35**±**3.60 | 3.26 |
|  | 3000 |  | 84.50**±**2.03 | 2.41 |  | 111.43**±**2.57 | 2.30 |
| Orn | 100 |  | 42.49**±**1.53 | 3.60 |  | 151.56**±**6.35 | 4.19 |
|  | 3000 |  | 61.42**±**1.09 | 1.77 |  | 117.00**±**5.20 | 4.45 |
| Ahd | 100 |  | 68.75**±**1.31 | 1.91 |  | 107.10**±**1.62 | 1.52 |
|  | 3000 |  | 106.72**±**2.61 | 2.45 |  | 96.57**±**1.88 | 1.95 |
| Hia | 100 |  | 111.56**±**1.75 | 1.57 |  | 104.91**±**2.24 | 2.14 |
|  | 3000 |  | 109.48**±**1.52 | 1.39 |  | 101.30**±**1.33 | 1.31 |
| SDMA | 100 |  | 63.76**±**3.85 | 6.04 |  | 95.76**±**6.73 | 7.03 |
|  | 3000 |  | 67.84**±**1.71 | 2.53 |  | 121.80**±**2.89 | 2.38 |
| Kyn | 100 |  | 86.39**±**2.03 | 2.35 |  | 120.02**±**4.11 | 3.43 |
|  | 3000 |  | 95.20**±**1.78 | 1.87 |  | 108.05**±**1.48 | 1.37 |
| L-Ala-d4 | 400 (QC1) |  | 87.00**±**1.71 | 1.97 |  | 83.36**±**2.00 | 2.40 |
|  | 400 (QC3) |  | 97.41**±**2.62 | 2.69 |  | 95.08**±**2.86 | 3.01 |
| L-Met-d3 | 400 (QC1) |  | 101.12**±**1.48 | 1.47 |  | 95.19**±**7.52 | 7.89 |
|  | 400 (QC3) |  | 79.73**±**2.26 | 2.84 |  | 134.26**±**1.51 | 1.13 |
| L-Phe-d5 | 400 (QC1) |  | 108.89**±**1.79 | 1.64 |  | 85.15**±**1.01 | 1.19 |
|  | 400 (QC3) |  | 115.68**±**2.28 | 1.97 |  | 89.86**±**1.19 | 1.32 |

**TABLE S3: The intra- and inter-day accuracy and precision of 34 amino acids (n=5).**

| **Analyte** | **Nominal  concn. (ng/ml)** |  | **Intra-day accuracy and precision** | | |  | **Inter-day accuracy and precision** | | |
| --- | --- | --- | --- | --- | --- | --- | --- | --- | --- |
|  |  |  | **Measured concn.  (ng/ml)** | **RE (%)** | **RSD (%)** |  | **Measured concn.  (ng/ml)** | **RE (%)** | **RSD (%)** |
| Gly | 1000 |  | 1011.77±36.27 | 1.18 | 3.58 |  | 1006.00±28.74 | 0.60 | 2.86 |
|  | 2000 |  | 1987.20±20.66 | -0.64 | 1.04 |  | 1979.38±39.04 | -1.03 | 1.97 |
|  | 10000 |  | 11349.10±112.96 | 13.49 | 1.00 |  | 11258.73±207.03 | 12.59 | 1.84 |
|  | 60000 |  | 58898.16±1615.73 | -1.84 | 2.74 |  | 57654.39±2330.16 | -3.91 | 4.04 |
| Ala | 1000 |  | 1055.33±6.74 | 5.53 | 0.64 |  | 1051.28±9.76 | 5.13 | 0.93 |
|  | 2000 |  | 1877.09±31.81 | -6.15 | 1.69 |  | 1887.14±18.12 | -5.64 | 0.96 |
|  | 10000 |  | 9422.01±73.65 | -5.78 | 0.78 |  | 9378.39±86.07 | -6.22 | 0.92 |
|  | 60000 |  | 63277.24±684.97 | 5.46 | 1.08 |  | 62766.22±1715.51 | 4.61 | 2.73 |
| Ser | 1000 |  | 829.88±18.06 | -17.01 | 2.18 |  | 867.26±96.82 | -13.27 | 11.16 |
|  | 2000 |  | 1794.52±103.51 | -10.27 | 5.77 |  | 1835.65±114.54 | -8.22 | 6.24 |
|  | 10000 |  | 10568.59±338.26 | 5.69 | 3.20 |  | 10150.30±435.02 | 1.50 | 4.29 |
|  | 60000 |  | 52229.51±931.03 | -12.95 | 1.78 |  | 51288.14±1234.12 | -14.52 | 2.41 |
| Val | 1000 |  | 984.73±16.93 | -1.53 | 1.72 |  | 969.59±51.11 | -3.04 | 5.27 |
|  | 2000 |  | 1833.26±57.85 | -8.34 | 3.16 |  | 1897.26±45.25 | -5.14 | 2.38 |
|  | 10000 |  | 10553.90±193.77 | 5.54 | 1.84 |  | 10285.73±107.79 | 2.86 | 1.05 |
|  | 60000 |  | 59673.11±2231.98 | -0.54 | 3.74 |  | 60994.49±1724.05 | 1.66 | 2.83 |
| Thr | 1000 |  | 1008.61±35.14 | 0.86 | 3.48 |  | 1000.60±26.81 | 0.06 | 2.68 |
|  | 2000 |  | 1890.83±26.95 | -5.46 | 1.43 |  | 1841.56±59.18 | -7.92 | 3.21 |
|  | 10000 |  | 9814.13±164.08 | -1.86 | 1.67 |  | 9773.11±113.85 | -2.27 | 1.16 |
|  | 60000 |  | 61016.84±2300.49 | 1.69 | 3.77 |  | 60445.75±2735.95 | 0.74 | 4.53 |
| Leu | 1000 |  | 1040.95±10.65 | 4.09 | 1.02 |  | 1035.84±36.83 | 3.58 | 3.56 |
|  | 2000 |  | 1891.09±23.62 | -5.45 | 1.25 |  | 1927.00±96.64 | -3.65 | 5.02 |
|  | 10000 |  | 9888.62±72.51 | -1.11 | 0.73 |  | 9980.13±114.26 | -0.20 | 1.14 |
|  | 60000 |  | 63227.83±2011.36 | 5.38 | 3.18 |  | 62027.60±1563.94 | 3.38 | 2.52 |
| Ile | 1000 |  | 1057.22±12.15 | 5.72 | 1.15 |  | 1056.39±27.24 | 5.64 | 2.58 |
|  | 2000 |  | 1951.53±20.49 | -2.42 | 1.05 |  | 1969.66±90.04 | -1.52 | 4.57 |
|  | 10000 |  | 9915.30±211.27 | -0.85 | 2.13 |  | 9991.94±169.78 | -0.08 | 1.70 |
|  | 60000 |  | 62279.19±884.24 | 3.80 | 1.42 |  | 62018.16±2138.01 | 3.36 | 3.45 |
| Asp | 1000 |  | 920.05±46.12 | -7.99 | 5.01 |  | 903.77±53.08 | -9.62 | 5.87 |
|  | 2000 |  | 2015.71±25.07 | 0.79 | 1.24 |  | 1919.51±141.33 | -4.02 | 7.36 |
|  | 10000 |  | 11027.73±258.25 | 10.28 | 2.34 |  | 10795.09±213.78 | 7.95 | 1.98 |
|  | 60000 |  | 59763.35±2374.95 | -0.39 | 3.97 |  | 58288.69±2447.55 | -2.85 | 4.20 |
| Lys | 1000 |  | 1090.81±31.48 | 9.08 | 2.89 |  | 1083.81±42.25 | 8.38 | 3.90 |
|  | 2000 |  | 1869.22±30.22 | -6.54 | 1.62 |  | 1850.59±79.27 | -7.47 | 4.28 |
|  | 10000 |  | 8827.82±98.34 | -11.72 | 1.11 |  | 9192.03±516.36 | -8.08 | 5.62 |
|  | 60000 |  | 67783.66±975.40 | 12.97 | 1.44 |  | 67068.06±1229.63 | 11.78 | 1.83 |
| Gln | 1000 |  | 924.62±34.22 | -7.54 | 3.70 |  | 913.66±35.16 | -8.63 | 3.85 |
|  | 2000 |  | 2199.42±27.62 | 9.97 | 1.26 |  | 2139.79±139.19 | 6.99 | 6.50 |
|  | 10000 |  | 10356.27±209.59 | 3.56 | 2.02 |  | 10042.38±311.27 | 0.42 | 3.10 |
|  | 60000 |  | 62188.25±1758.30 | 3.65 | 2.83 |  | 60898.90±1883.16 | 1.50 | 3.09 |
| Glu | 1000 |  | 974.86±28.40 | -2.51 | 2.91 |  | 974.85±23.70 | -2.51 | 2.43 |
|  | 2000 |  | 2027.68±43.80 | 1.38 | 2.16 |  | 1980.58±85.00 | -0.97 | 4.29 |
|  | 10000 |  | 10247.89±122.28 | 2.48 | 1.19 |  | 10179.70±202.52 | 1.80 | 1.99 |
|  | 60000 |  | 59515.43±1434.97 | -0.81 | 2.41 |  | 58690.85±2407.68 | -2.18 | 4.10 |
| Phe | 1000 |  | 1121.80±11.32 | 12.18 | 1.01 |  | 1129.54±34.84 | 12.95 | 3.08 |
|  | 2000 |  | 1928.13±19.26 | -3.59 | 1.00 |  | 1929.82±87.11 | -3.51 | 4.51 |
|  | 10000 |  | 10355.83±85.03 | 3.56 | 0.82 |  | 10371.74±206.11 | 3.72 | 1.99 |
|  | 60000 |  | 65902.58±1854.85 | 9.84 | 2.81 |  | 64068.64±1764.70 | 6.78 | 2.75 |
| Arg | 1000 |  | 957.42±37.44 | -4.26 | 3.91 |  | 970.06±27.92 | -2.99 | 2.88 |
|  | 2000 |  | 2067.01±41.96 | 3.35 | 2.03 |  | 2016.78±81.73 | 0.84 | 4.05 |
|  | 10000 |  | 9847.64±203.99 | -1.52 | 2.07 |  | 9853.47±96.25 | -1.47 | 0.98 |
|  | 60000 |  | 64025.45±1401.41 | 6.71 | 2.19 |  | 63553.46±1743.36 | 5.92 | 2.74 |
| Tyr | 1000 |  | 967.57±68.10 | -3.24 | 7.04 |  | 940.45±89.56 | -5.95 | 9.52 |
|  | 2000 |  | 2070.86±113.91 | 3.54 | 5.50 |  | 2004.96±197.66 | 0.25 | 9.86 |
|  | 10000 |  | 9059.26±71.78 | -9.41 | 0.79 |  | 9121.09±387.55 | -8.79 | 4.25 |
|  | 60000 |  | 58724.64±1989.46 | -2.13 | 3.39 |  | 59687.05±2031.82 | -0.52 | 3.40 |
| Pro | 500 |  | 584.97±25.81 | 16.99 | 4.41 |  | 579.72±17.72 | 15.94 | 3.06 |
|  | 1000 |  | 1100.14±6.57 | 10.01 | 0.60 |  | 1031.13±36.24 | 3.11 | 3.51 |
|  | 5000 |  | 5710.46±33.57 | 14.21 | 0.59 |  | 5345.90±290.18 | 6.92 | 5.43 |
|  | 30000 |  | 33124.21±1108.64 | 10.41 | 3.35 |  | 33250.77±526.92 | 10.84 | 1.58 |
| Asn | 500 |  | 433.28±27.76 | -13.34 | 6.41 |  | 437.19±28.33 | -12.56 | 6.48 |
|  | 1000 |  | 1077.78±10.98 | 7.78 | 1.02 |  | 1047.10±67.19 | 4.71 | 6.42 |
|  | 5000 |  | 5145.84±143.67 | 2.92 | 2.79 |  | 5148.70±129.96 | 2.97 | 2.52 |
|  | 30000 |  | 30287.64±1464.88 | 0.96 | 4.84 |  | 29819.93±1293.00 | -0.60 | 4.34 |
| Met | 500 |  | 514.55±2.93 | 2.91 | 0.57 |  | 516.14±5.38 | 3.23 | 1.04 |
|  | 1000 |  | 929.40±8.56 | -7.06 | 0.92 |  | 939.03±15.57 | -6.10 | 1.66 |
|  | 5000 |  | 4638.77±20.70 | -7.22 | 0.45 |  | 4633.77±45.68 | -7.32 | 0.99 |
|  | 30000 |  | 33454.08±409.49 | 11.51 | 1.22 |  | 32966.67±1321.44 | 9.89 | 4.01 |
| Trp | 500 |  | 499.94±11.73 | -0.01 | 2.35 |  | 497.55±37.95 | -0.49 | 7.63 |
|  | 1000 |  | 927.27±20.41 | -7.27 | 2.20 |  | 908.53±30.78 | -9.15 | 3.39 |
|  | 5000 |  | 4634.10±28.53 | -7.32 | 0.62 |  | 4589.92±158.94 | -8.20 | 3.46 |
|  | 30000 |  | 31140.32±241.21 | 3.80 | 0.77 |  | 30960.33±927.06 | 3.20 | 2.99 |
| Cys | 100 |  | 91.52±4.81 | -8.48 | 5.26 |  | 85.37±5.83 | -14.63 | 6.83 |
|  | 200 |  | 176.77±2.86 | -11.61 | 1.62 |  | 179.10±8.63 | -10.45 | 4.82 |
|  | 1000 |  | 1042.16±28.15 | 4.22 | 2.70 |  | 1017.20±39.10 | 1.72 | 3.84 |
|  | 6000 |  | 5744.74±176.38 | -4.25 | 3.07 |  | 5615.25±237.50 | -6.41 | 4.23 |
| His | 100 |  | 86.94±3.36 | -13.06 | 3.86 |  | 86.30±4.03 | -13.70 | 4.67 |
|  | 200 |  | 223.86±2.83 | 11.93 | 1.27 |  | 219.89±12.07 | 9.94 | 5.49 |
|  | 1000 |  | 1119.36±10.12 | 11.94 | 0.90 |  | 1116.14±24.05 | 11.61 | 2.15 |
|  | 6000 |  | 5504.09±191.04 | -8.27 | 3.47 |  | 5565.00±228.76 | -7.25 | 4.11 |
| Cit | 100 |  | 100.44±4.19 | 0.44 | 4.17 |  | 103.43±4.13 | 3.43 | 4.00 |
|  | 200 |  | 217.91±7.10 | 8.95 | 3.26 |  | 198.72±20.49 | -0.64 | 10.31 |
|  | 1000 |  | 904.85±44.07 | -9.52 | 4.87 |  | 934.93±72.37 | -6.51 | 7.74 |
|  | 6000 |  | 5754.51±280.90 | -4.09 | 4.88 |  | 5361.67±321.67 | -10.64 | 6.00 |
| ADMA | 100 |  | 92.54±7.03 | -7.46 | 7.60 |  | 93.59±7.09 | -6.41 | 7.57 |
|  | 200 |  | 174.37±1.64 | -12.82 | 0.94 |  | 177.88±8.30 | -11.06 | 4.67 |
|  | 1000 |  | 985.73±17.45 | -1.43 | 1.77 |  | 1000.58±28.74 | 0.06 | 2.87 |
|  | 6000 |  | 5421.21±149.99 | -9.65 | 2.77 |  | 5412.95±156.90 | -9.78 | 2.90 |
| Cyss | 100 |  | 113.47±4.93 | 13.47 | 4.35 |  | 115.05±3.50 | 15.05 | 3.04 |
|  | 200 |  | 214.78±12.56 | 7.39 | 5.85 |  | 204.24±15.91 | 2.12 | 7.79 |
|  | 1000 |  | 1040.33±24.16 | 4.03 | 2.32 |  | 1032.42±70.01 | 3.24 | 6.78 |
|  | 6000 |  | 5523.21±197.63 | -7.95 | 3.58 |  | 5495.32±177.70 | -8.41 | 3.23 |
| Sar | 50 |  | 50.76±1.66 | 1.52 | 3.27 |  | 50.77±1.60 | 1.53 | 3.16 |
|  | 100 |  | 92.23±2.56 | -7.77 | 2.77 |  | 93.23±6.00 | -6.77 | 6.43 |
|  | 500 |  | 519.92±10.01 | 3.98 | 1.93 |  | 520.82±6.45 | 4.16 | 1.24 |
|  | 3000 |  | 3035.15±121.53 | 1.17 | 4.00 |  | 2978.35±116.99 | -0.72 | 3.93 |
| Apa | 50 |  | 51.05±2.96 | 2.11 | 5.81 |  | 50.94±2.58 | 1.88 | 5.07 |
|  | 100 |  | 99.05±7.54 | -0.95 | 7.61 |  | 96.99±5.50 | -3.01 | 5.67 |
|  | 500 |  | 500.92±7.46 | 0.18 | 1.49 |  | 480.50±15.40 | -3.90 | 3.20 |
|  | 3000 |  | 3269.64±105.25 | 8.99 | 3.22 |  | 3170.47±75.67 | 5.68 | 2.39 |
| Amp | 50 |  | 41.66±1.01 | -16.69 | 2.43 |  | 42.44±4.48 | -15.13 | 10.57 |
|  | 100 |  | 98.73±4.36 | -1.27 | 4.42 |  | 97.50±6.75 | -2.50 | 6.92 |
|  | 500 |  | 529.23±9.43 | 5.85 | 1.78 |  | 517.40±26.49 | 3.48 | 5.12 |
|  | 3000 |  | 2631.92±27.27 | -12.27 | 1.04 |  | 2630.50±86.46 | -12.32 | 3.29 |
| Aba | 50 |  | 46.86±1.23 | -6.28 | 2.62 |  | 47.21±1.15 | -5.58 | 2.45 |
|  | 100 |  | 96.80±1.95 | -3.20 | 2.02 |  | 96.70±5.32 | -3.30 | 5.50 |
|  | 500 |  | 523.80±9.17 | 4.76 | 1.75 |  | 523.82±8.91 | 4.76 | 1.70 |
|  | 3000 |  | 3004.07±27.48 | 0.14 | 0.91 |  | 2980.85±119.74 | -0.64 | 4.02 |
| Opr | 50 |  | 49.62±4.79 | -0.75 | 9.66 |  | 49.82±4.72 | -0.36 | 9.47 |
|  | 100 |  | 106.14±5.97 | 6.14 | 5.62 |  | 98.10±8.44 | -1.90 | 8.61 |
|  | 500 |  | 483.74±41.47 | -3.25 | 8.57 |  | 489.89±33.02 | -2.02 | 6.74 |
|  | 3000 |  | 2979.94±230.91 | -0.67 | 7.75 |  | 3233.87±161.42 | 7.80 | 4.99 |
| Hpr | 50 |  | 48.90±2.62 | -2.20 | 5.36 |  | 49.78±2.98 | -0.44 | 5.98 |
|  | 100 |  | 101.83±2.58 | 1.83 | 2.53 |  | 97.43±4.10 | -2.57 | 4.20 |
|  | 500 |  | 511.72±4.45 | 2.34 | 0.87 |  | 508.57±8.02 | 1.71 | 1.58 |
|  | 3000 |  | 3110.02±128.08 | 3.67 | 4.12 |  | 3065.24±118.80 | 2.17 | 3.88 |
| Orn | 50 |  | 41.14±0.52 | -17.71 | 1.26 |  | 40.99±0.72 | -18.03 | 1.75 |
|  | 100 |  | 109.34±1.70 | 9.34 | 1.55 |  | 104.05±10.04 | 4.05 | 9.65 |
|  | 500 |  | 549.66±14.84 | 9.93 | 2.70 |  | 559.77±11.36 | 11.95 | 2.03 |
|  | 3000 |  | 2705.51±103.18 | -9.82 | 3.81 |  | 2672.19±127.85 | -10.93 | 4.78 |
| Ahd | 50 |  | 46.03±1.12 | -7.93 | 2.44 |  | 44.50±1.74 | -11.00 | 3.92 |
|  | 100 |  | 101.99±2.08 | 1.99 | 2.04 |  | 102.33±3.77 | 2.33 | 3.68 |
|  | 500 |  | 499.72±7.07 | -0.06 | 1.41 |  | 489.63±15.49 | -2.07 | 3.16 |
|  | 3000 |  | 2997.87±225.18 | -0.07 | 7.51 |  | 3126.23±162.02 | 4.21 | 5.18 |
| Hia | 50 |  | 48.18±0.60 | -3.64 | 1.24 |  | 47.32±2.23 | -5.36 | 4.71 |
|  | 100 |  | 97.56±3.50 | -2.44 | 3.59 |  | 97.43±1.91 | -2.57 | 1.96 |
|  | 500 |  | 520.66±4.68 | 4.13 | 0.90 |  | 523.04±11.93 | 4.61 | 2.28 |
|  | 3000 |  | 2805.94±58.97 | -6.47 | 2.10 |  | 2789.87±66.88 | -7.00 | 2.40 |
| SDMA | 50 |  | 43.12±5.71 | -13.77 | 13.25 |  | 48.16±5.93 | -3.69 | 12.32 |
|  | 100 |  | 86.48±1.32 | -13.52 | 1.52 |  | 90.56±5.80 | -9.44 | 6.41 |
|  | 500 |  | 463.27±10.95 | -7.35 | 2.36 |  | 461.72±26.18 | -7.66 | 5.67 |
|  | 3000 |  | 2784.82±73.66 | -7.17 | 2.65 |  | 2760.45±82.81 | -7.98 | 3.00 |
| Kyn | 50 |  | 55.03±2.18 | 10.06 | 3.95 |  | 53.98±4.50 | 7.97 | 8.34 |
|  | 100 |  | 100.73±1.78 | 0.73 | 1.77 |  | 100.01±2.91 | 0.01 | 2.91 |
|  | 500 |  | 461.45±4.01 | -7.71 | 0.87 |  | 477.03±11.82 | -4.59 | 2.48 |
|  | 3000 |  | 3047.42±65.64 | 1.58 | 2.15 |  | 3081.60±65.29 | 2.72 | 2.12 |

**TABLE S4: The stability results of 34 amino acids (n=3).**

| **Analyte** | **Nominal concn.**  **(ng/ml)** |  | **room temperature stability** | |  | **Freeze-thaw stability** | |  | **Short-term stability** | |  | **Long-term stability** | |
| --- | --- | --- | --- | --- | --- | --- | --- | --- | --- | --- | --- | --- | --- |
|  |  |  | **RE (%)** | **RSD (%)** |  | **RE (%)** | **RSD (%)** |  | **RE (%)** | **RSD (%)** |  | **RE (%)** | **RSD (%)** |
| Gly | 2000 |  | -1.93 | 2.68 |  | -0.94 | 1.32 |  | -3.77 | 2.11 |  | -1.20 | 3.59 |
|  | 10000 |  | 12.23 | 2.19 |  | 13.45 | 1.00 |  | 10.04 | 0.66 |  | 12.55 | 1.79 |
|  | 60000 |  | -6.54 | 2.86 |  | -1.04 | 3.50 |  | -8.84 | 1.47 |  | -6.28 | 2.48 |
| Ala | 2000 |  | -5.45 | 3.04 |  | -5.93 | 1.80 |  | -5.53 | 3.78 |  | -5.52 | 2.44 |
|  | 10000 |  | -6.39 | 1.95 |  | -6.00 | 1.01 |  | -6.82 | 2.44 |  | -6.81 | 1.73 |
|  | 60000 |  | 3.41 | 3.33 |  | 5.51 | 1.09 |  | 1.79 | 1.64 |  | 4.00 | 2.84 |
| Ser | 2000 |  | -1.26 | 8.41 |  | -10.81 | 6.03 |  | 4.64 | 1.18 |  | -3.97 | 8.32 |
|  | 10000 |  | 1.73 | 5.75 |  | 6.09 | 3.35 |  | 6.02 | 1.34 |  | 1.99 | 4.59 |
|  | 60000 |  | -12.88 | 1.92 |  | -13.24 | 2.01 |  | -11.87 | 1.89 |  | -13.35 | 1.75 |
| Val | 2000 |  | -5.93 | 4.28 |  | -7.63 | 3.77 |  | -4.71 | 2.25 |  | -7.15 | 4.42 |
|  | 10000 |  | 1.97 | 2.80 |  | 4.98 | 2.36 |  | 1.51 | 3.48 |  | 2.02 | 2.64 |
|  | 60000 |  | 0.66 | 2.94 |  | -1.28 | 4.29 |  | -0.91 | 2.27 |  | 0.44 | 2.38 |
| Thr | 2000 |  | -7.08 | 1.62 |  | -5.61 | 1.50 |  | -6.68 | 1.57 |  | -6.55 | 1.60 |
|  | 10000 |  | -3.42 | 3.06 |  | -1.69 | 1.73 |  | -5.47 | 1.78 |  | -2.95 | 2.67 |
|  | 60000 |  | -1.42 | 0.58 |  | 2.79 | 4.74 |  | -1.65 | 0.73 |  | -1.52 | 0.79 |
| Leu | 2000 |  | -5.78 | 0.80 |  | 0.32 | 2.10 |  | -6.19 | 0.95 |  | -5.06 | 1.32 |
|  | 10000 |  | -1.84 | 1.68 |  | -7.55 | 0.99 |  | -2.59 | 1.10 |  | -0.99 | 1.91 |
|  | 60000 |  | 1.15 | 2.25 |  | 1.19 | 2.40 |  | -0.42 | 2.18 |  | 0.96 | 1.90 |
| Ile | 2000 |  | -5.67 | 0.80 |  | -8.25 | 1.12 |  | -6.03 | 0.09 |  | -4.85 | 1.47 |
|  | 10000 |  | -1.58 | 1.58 |  | 2.53 | 2.10 |  | -1.81 | 2.17 |  | -1.12 | 1.61 |
|  | 60000 |  | 0.06 | 3.52 |  | 6.78 | 5.21 |  | -2.02 | 4.22 |  | -0.11 | 2.85 |
| Asp | 2000 |  | -2.83 | 5.38 |  | 0.67 | 1.28 |  | -0.01 | 1.03 |  | -3.47 | 4.62 |
|  | 10000 |  | 6.24 | 2.39 |  | 10.03 | 2.43 |  | 4.40 | 0.29 |  | 6.17 | 2.25 |
|  | 60000 |  | -4.23 | 0.67 |  | 0.77 | 5.04 |  | -4.64 | 0.36 |  | -4.94 | 1.94 |
| Lys | 2000 |  | -2.28 | 4.15 |  | -6.90 | 1.94 |  | 1.20 | 0.91 |  | -3.69 | 4.07 |
|  | 10000 |  | -12.88 | 1.53 |  | -11.68 | 1.12 |  | -12.90 | 1.41 |  | -12.85 | 1.37 |
|  | 60000 |  | 11.64 | 0.95 |  | 12.67 | 1.62 |  | 11.96 | 0.30 |  | 11.25 | 1.54 |
| Gln | 2000 |  | 8.54 | 1.99 |  | 9.89 | 1.27 |  | 8.42 | 0.95 |  | 8.62 | 1.78 |
|  | 10000 |  | 0.86 | 1.96 |  | 3.65 | 2.04 |  | -0.26 | 0.87 |  | 0.23 | 2.06 |
|  | 60000 |  | 0.60 | 1.07 |  | 4.52 | 3.62 |  | -0.30 | 0.66 |  | -0.06 | 1.40 |
| Glu | 2000 |  | -1.53 | 3.56 |  | 0.86 | 2.60 |  | -0.28 | 1.89 |  | -0.39 | 3.36 |
|  | 10000 |  | 0.09 | 2.84 |  | 2.57 | 1.22 |  | -1.76 | 1.01 |  | 0.56 | 2.38 |
|  | 60000 |  | -4.30 | 1.72 |  | -0.12 | 3.06 |  | -5.13 | 0.85 |  | -4.53 | 1.49 |
| Phe | 2000 |  | -7.17 | 1.65 |  | -2.37 | 3.59 |  | -8.30 | 1.02 |  | -5.97 | 2.39 |
|  | 10000 |  | 1.71 | 2.59 |  | 3.85 | 1.16 |  | -0.12 | 1.88 |  | 1.92 | 2.14 |
|  | 60000 |  | 4.17 | 2.90 |  | -6.92 | 2.50 |  | 1.84 | 2.40 |  | 3.85 | 2.35 |
| Arg | 2000 |  | 2.47 | 2.05 |  | 2.73 | 2.63 |  | 3.73 | 0.38 |  | 3.38 | 2.35 |
|  | 10000 |  | -2.94 | 1.27 |  | -1.17 | 2.29 |  | -3.23 | 0.94 |  | -2.51 | 1.41 |
|  | 60000 |  | 1.76 | 2.56 |  | 7.35 | 2.73 |  | -0.06 | 1.97 |  | 2.97 | 2.79 |
| Tyr | 2000 |  | -5.11 | 6.83 |  | 3.28 | 5.56 |  | 0.38 | 1.34 |  | -6.92 | 6.69 |
|  | 10000 |  | -7.14 | 5.95 |  | -9.40 | 0.79 |  | -4.20 | 6.39 |  | -9.00 | 5.70 |
|  | 60000 |  | 3.16 | 2.23 |  | -2.97 | 4.17 |  | 4.58 | 1.15 |  | 1.04 | 3.82 |
| Pro | 1000 |  | 2.16 | 5.18 |  | 10.21 | 0.76 |  | -1.66 | 5.11 |  | 2.34 | 4.11 |
|  | 5000 |  | 10.63 | 3.80 |  | 14.01 | 0.75 |  | 13.04 | 0.95 |  | 10.94 | 3.09 |
|  | 30000 |  | 11.77 | 1.53 |  | 9.53 | 4.04 |  | 12.70 | 1.52 |  | 11.91 | 1.46 |
| Asn | 1000 |  | 7.45 | 4.54 |  | 7.67 | 1.06 |  | 8.84 | 2.13 |  | 7.17 | 3.67 |
|  | 5000 |  | 0.89 | 3.90 |  | 2.57 | 2.95 |  | -1.56 | 1.50 |  | 0.46 | 3.39 |
|  | 30000 |  | -2.94 | 1.87 |  | 2.30 | 5.97 |  | -4.48 | 0.75 |  | -3.41 | 2.04 |
| Met | 1000 |  | -6.83 | 1.33 |  | -6.83 | 1.14 |  | -6.84 | 1.84 |  | -6.44 | 1.28 |
|  | 5000 |  | -7.86 | 1.33 |  | -7.21 | 0.45 |  | -7.69 | 1.67 |  | -7.86 | 1.33 |
|  | 30000 |  | 11.97 | 1.21 |  | 11.13 | 1.54 |  | 12.07 | 1.82 |  | 11.13 | 1.50 |
| Trp | 1000 |  | -5.29 | 4.17 |  | -3.81 | 1.18 |  | -2.72 | 1.85 |  | -6.74 | 4.12 |
|  | 5000 |  | -6.93 | 2.87 |  | 3.72 | 0.93 |  | -5.20 | 2.47 |  | -7.86 | 2.76 |
|  | 30000 |  | 3.53 | 0.95 |  | 10.56 | 3.32 |  | 3.96 | 1.05 |  | 3.33 | 1.29 |
| Cys | 200 |  | -10.21 | 1.30 |  | -12.02 | 2.05 |  | -10.08 | 0.59 |  | -11.40 | 2.35 |
|  | 1000 |  | 2.79 | 1.75 |  | 4.03 | 2.75 |  | 2.32 | 2.18 |  | 0.41 | 4.10 |
|  | 6000 |  | -7.12 | 1.16 |  | -3.38 | 3.93 |  | -6.98 | 1.22 |  | -8.06 | 1.96 |
| His | 200 |  | 11.89 | 1.67 |  | 11.62 | 1.48 |  | 12.69 | 0.19 |  | 12.36 | 1.48 |
|  | 1000 |  | 10.13 | 1.82 |  | 12.02 | 0.92 |  | 9.52 | 2.11 |  | 10.02 | 1.79 |
|  | 6000 |  | -9.22 | 0.76 |  | -9.06 | 4.24 |  | -9.71 | 0.52 |  | -9.29 | 1.13 |
| Cit | 200 |  | 6.91 | 9.30 |  | 9.57 | 3.59 |  | 9.35 | 2.11 |  | 1.23 | 11.60 |
|  | 1000 |  | -6.95 | 2.47 |  | -9.48 | 4.87 |  | -6.65 | 3.41 |  | -8.59 | 3.36 |
|  | 6000 |  | -5.98 | 4.26 |  | -2.75 | 6.12 |  | -5.62 | 5.68 |  | -6.67 | 4.88 |
| ADMA | 200 |  | -9.40 | 4.88 |  | -12.98 | 1.08 |  | -6.85 | 5.69 |  | -10.02 | 4.04 |
|  | 1000 |  | -4.27 | 5.17 |  | -1.09 | 2.00 |  | -4.70 | 7.95 |  | -3.15 | 4.48 |
|  | 6000 |  | -9.05 | 2.56 |  | -10.24 | 3.32 |  | -7.52 | 1.42 |  | -10.14 | 2.73 |
| Cyss | 200 |  | 3.35 | 9.21 |  | 5.56 | 7.60 |  | 5.05 | 9.89 |  | 3.40 | 7.66 |
|  | 1000 |  | 4.88 | 3.30 |  | 4.24 | 2.38 |  | 6.38 | 2.51 |  | 4.70 | 2.97 |
|  | 6000 |  | -6.77 | 2.23 |  | -8.87 | 4.56 |  | -5.32 | 0.79 |  | -8.02 | 2.72 |
| Sar | 100 |  | -9.92 | 3.02 |  | -8.44 | 3.43 |  | -11.26 | 3.35 |  | -8.93 | 3.47 |
|  | 500 |  | 2.30 | 1.68 |  | 4.27 | 2.06 |  | 0.89 | 0.08 |  | 3.28 | 2.03 |
|  | 3000 |  | -2.66 | 1.14 |  | 2.38 | 5.11 |  | -3.57 | 0.67 |  | -2.60 | 1.26 |
| Apa | 100 |  | -4.58 | 3.00 |  | -1.42 | 7.76 |  | -5.70 | 4.20 |  | -3.63 | 3.11 |
|  | 500 |  | -5.05 | 2.69 |  | -0.26 | 1.93 |  | -6.10 | 3.31 |  | -4.58 | 2.33 |
|  | 3000 |  | 4.58 | 2.18 |  | 9.40 | 3.37 |  | 3.98 | 2.95 |  | 4.60 | 2.25 |
| Amp | 100 |  | 3.42 | 3.58 |  | -2.09 | 5.02 |  | 4.88 | 1.08 |  | 2.18 | 3.62 |
|  | 500 |  | 2.24 | 3.12 |  | 5.32 | 2.25 |  | 3.91 | 3.92 |  | 2.28 | 2.49 |
|  | 3000 |  | -12.39 | 0.91 |  | -12.03 | 1.28 |  | -12.21 | 1.10 |  | -12.93 | 1.22 |
| Aba | 100 |  | -3.71 | 1.49 |  | -3.26 | 2.03 |  | -3.95 | 1.74 |  | -3.57 | 1.40 |
|  | 500 |  | 3.69 | 1.97 |  | 4.91 | 1.79 |  | 3.80 | 2.27 |  | 2.94 | 2.30 |
|  | 3000 |  | -2.67 | 0.82 |  | 0.36 | 1.10 |  | -3.22 | 0.70 |  | -2.95 | 1.61 |
| Opr | 100 |  | -1.79 | 5.01 |  | 5.46 | 5.92 |  | -5.47 | 2.67 |  | -2.92 | 5.88 |
|  | 500 |  | -7.49 | 5.11 |  | -2.96 | 8.59 |  | -5.40 | 5.12 |  | -4.11 | 6.66 |
|  | 3000 |  | 1.15 | 6.92 |  | -2.65 | 9.68 |  | -5.06 | 2.56 |  | 4.57 | 7.26 |
| Hpr | 100 |  | 2.20 | 4.10 |  | 2.52 | 3.13 |  | 1.57 | 2.30 |  | 2.38 | 3.45 |
|  | 500 |  | 1.01 | 3.33 |  | 2.31 | 0.87 |  | -0.87 | 1.34 |  | 0.89 | 2.70 |
|  | 3000 |  | -0.87 | 2.04 |  | 4.84 | 5.10 |  | -1.99 | 2.56 |  | -0.92 | 1.72 |
| Orn | 100 |  | 1.51 | 6.17 |  | 9.85 | 1.99 |  | 0.20 | 5.78 |  | 2.87 | 6.05 |
|  | 500 |  | 11.55 | 1.88 |  | 9.65 | 2.79 |  | 11.74 | 2.08 |  | 11.88 | 1.60 |
|  | 3000 |  | -13.65 | 0.74 |  | -8.94 | 4.61 |  | -13.82 | 0.36 |  | -12.89 | 2.40 |
| Ahd | 100 |  | 2.75 | 2.85 |  | 2.00 | 2.04 |  | 4.53 | 3.28 |  | 1.54 | 2.95 |
|  | 500 |  | -1.86 | 2.32 |  | -0.16 | 1.44 |  | -0.39 | 2.53 |  | -2.80 | 2.68 |
|  | 3000 |  | 6.50 | 3.58 |  | -2.30 | 9.91 |  | 7.29 | 4.09 |  | 5.90 | 3.13 |
| Hia | 100 |  | -1.56 | 2.20 |  | -5.57 | 1.31 |  | -0.45 | 2.80 |  | -3.33 | 3.28 |
|  | 500 |  | 5.20 | 1.35 |  | -1.11 | 0.73 |  | 6.15 | 1.44 |  | 3.62 | 2.53 |
|  | 3000 |  | -5.74 | 1.24 |  | 6.29 | 3.94 |  | -5.48 | 1.71 |  | -7.21 | 2.64 |
| SDMA | 100 |  | -7.97 | 6.36 |  | -13.13 | 1.95 |  | -5.40 | 4.08 |  | -9.83 | 6.00 |
|  | 500 |  | -6.30 | 5.32 |  | -7.29 | 2.37 |  | -2.25 | 2.61 |  | -6.36 | 4.53 |
|  | 3000 |  | -5.34 | 3.95 |  | -7.87 | 3.38 |  | -2.42 | 1.83 |  | -6.86 | 4.05 |
| Kyn | 100 |  | -1.01 | 2.17 |  | -2.56 | 1.12 |  | 0.82 | 0.94 |  | -1.58 | 1.94 |
|  | 500 |  | -4.14 | 3.22 |  | -0.96 | 2.16 |  | -1.70 | 2.38 |  | -4.72 | 2.82 |
|  | 3000 |  | 5.36 | 2.14 |  | 4.07 | 1.58 |  | 7.01 | 1.12 |  | 3.44 | 3.35 |

**TABLE S5: The dilution effect results of 34 amino acids (n=3).**

| **Analyte** |  | **8 fold dilution** | | |
| --- | --- | --- | --- | --- |
|  |  | **Measured concn. (ng/ml)** | **RE (%)** | **RSD (%)** |
| Gly |  | 11441.75±41.68 | 14.42 | 0.36 |
| Ala |  | 9403.85±163.29 | -5.96 | 1.74 |
| Ser |  | 9744.33±531.84 | -2.56 | 5.46 |
| Val |  | 10242.43±268.72 | 2.42 | 2.62 |
| Thr |  | 9862.83±254.07 | -1.37 | 2.58 |
| Leu |  | 9891.78±198.59 | -1.08 | 2.01 |
| Ile |  | 9864.42±114.62 | -1.36 | 1.16 |
| Asp |  | 10807.92±243.14 | 8.08 | 2.25 |
| Lys |  | 8714.32±171.40 | -12.86 | 1.97 |
| Gln |  | 10198.57±227.90 | 1.99 | 2.23 |
| Glu |  | 10194.74±297.29 | 1.95 | 2.92 |
| Phe |  | 10354.42±193.85 | 3.54 | 1.87 |
| Arg |  | 9734.70±164.72 | -2.65 | 1.69 |
| Tyr |  | 8992.42±361.38 | -10.08 | 4.02 |
| Pro |  | 5411.04±252.94 | 8.22 | 4.67 |
| Asn |  | 5167.13±215.13 | 3.34 | 4.16 |
| Met |  | 4598.74±56.91 | -8.03 | 1.24 |
| Trp |  | 4566.73±91.31 | -8.67 | 2.00 |
| Cys |  | 1032.51±15.59 | 3.25 | 1.51 |
| His |  | 1107.33±18.97 | 10.73 | 1.71 |
| Cit |  | 927.47±16.52 | -7.25 | 1.78 |
| ADMA |  | 961.67±17.94 | -3.83 | 1.87 |
| Cyss |  | 1033.85±40.05 | 3.38 | 3.87 |
| Sar |  | 518.54±5.91 | 3.71 | 1.14 |
| Apa |  | 480.04±9.01 | -3.99 | 1.88 |
| Amp |  | 502.86±3.38 | 0.57 | 0.67 |
| Aba |  | 517.92±11.09 | 3.58 | 2.14 |
| Opr |  | 452.08±21.99 | -9.58 | 4.86 |
| Hpr |  | 514.41±19.99 | 2.88 | 3.89 |
| Orn |  | 556.79±11.76 | 11.36 | 2.11 |
| Ahd |  | 483.36±1.81 | -3.33 | 0.37 |
| Hia |  | 521.25±1.17 | 4.25 | 0.22 |
| SDMA |  | 448.23±12.68 | -10.35 | 2.83 |
| Kyn |  | 467.10±3.48 | -6.58 | 0.74 |

|   **TIC** |   **TIC** |   **TIC** |
| --- | --- | --- |
|  |   Gly |   Gly |
|  |   ←Ala |   ←Ala |
|  |   Ser |   Ser |
|  |   ←Val |   ←Val |
|  |   Thr |   Thr |
|  |   ←Leu |   ←Leu |
|  |   Asp |   Asp |
|  |   Ile→ |   Ile→ |
|  |   ←Lys |   ←Lys |
|  |   ←Gln  ←Gln |  |
|  |   Glu |   Glu |
|  |   0  Phe |   Phe |
|  |   Arg |   Arg |
|  |   Tyr |   Tyr |
|  |   ←Pro |   ←Pro |
|  |   Asn |   Asn |
|  |   ←Met |   ←Met |
|  |   Trp |   Trp |
|  |   Cys |   Cys |
|  |   His |   His |
|  | ←Cit | ←Cit |
|  | ←ADMA | ←ADMA |
|  | Cyss | Cyss |
|  | sar→ | sar→ |
|  | Apa | Apa |
|  | ←Amp | ←Amp |
|  | Aba | Aba |
|  | ←Opr | ←Opr |
|  | ←Hpr | ←Hpr |
|  | ←Orn | ←Orn |
|  | Ahd | Ahd |
|  | Hia | Hia |
|  | ←SDMA | ←SDMA |
|  | Kyn | Kyn |
|  | L-Ala-d4 | L-Ala-d4 |
|  | ←L-Met-d3 | ←L-Met-d3 |
|  | L-Phe-d5 | L-Phe-d5 |
| **(a)** | **(b)** | **(c)** |

**FIGURE S1: The representative total ion current chromatograms and MRM chromatograms** **of 34 amino acids and 3 ISs. (a) blank matrix; (b) blank matrix spiked with 34 amino acid and 3 ISs; (c) cancerous tissue sample.**
